# Childhood inflammatory markers and intelligence as predictors of subsequent persistent depressive symptoms: a longitudinal cohort study

**DOI:** 10.1017/S0033291717003038

**Published:** 2017-11-15

**Authors:** G. M. Khandaker, J. Stochl, S. Zammit, I. Goodyer, G. Lewis, P. B. Jones

**Affiliations:** 1 Department of Psychiatry, University of Cambridge, Cambridge, UK; 2 Cambridgeshire and Peterborough NHS Foundation Trust, Cambridge, UK; 3 Department of Kinanthropology, Charles University, Prague, Czech Republic; 4 Centre for Mental Health, Addiction and Suicide Research, School of Social and Community Medicine, University of Bristol, Bristol, UK; 5 Institute of Psychological Medicine and Clinical Neurosciences, MRC Centre for Neuropsychiatric Genetics and Genomics, Cardiff University, Cardiff, UK; 6 Division of Psychiatry, University College London, London, UK

**Keywords:** CRP, depression, depressive symptoms, IL-6, immunopsychiatry, inflammation, intelligence, IQ, latent class analysis, longitudinal study, neurodevelopment

## Abstract

**Background:**

To identify developmental sub-groups of depressive symptoms during the second decade of life, a critical period of brain development, using data from a prospective birth cohort. To test whether childhood intelligence and inflammatory markers are associated with subsequent persistent depressive symptoms.

**Methods:**

IQ, a proxy for neurodevelopment, was measured at age 8 years. Interleukin 6 (IL-6) and C-reactive protein, typical inflammatory markers, were measured at age 9 years. Depressive symptoms were measured six times between 10 and 19 years using the short mood and feelings questionnaire (SMFQ), which were coded as binary variable and then used in latent class analysis to identify developmental sub-groups of depressive symptoms.

**Results:**

Longitudinal SMFQ data from 9156 participants yielded three distinct population sub-groups of depressive symptoms: no symptoms (81.2%); adolescent-onset symptoms (13.2%); persistent symptoms (5.6%). Lower IQ and higher IL-6 levels in childhood were independently associated with subsequent persistent depressive symptoms in a linear, dose–response fashion, but not with adolescent-onset symptoms. Compared with the group with no symptoms the adjusted odds ratio for persistent depressive symptoms per s.d. increase in IQ was 0.80 (95% CI, 0.68–0.95); that for IL-6 was 1.20 (95% CI, 1.03–1.39). Evidence for an association with IL-6 remained after controlling for initial severity of depressive symptoms at 10 years. There was no evidence that IL-6 moderated or mediated the IQ-persistent depressive symptom relationship.

**Conclusions:**

The results indicate potentially important roles for two distinct biological processes, neurodevelopment and inflammation, in the aetiology of persistent depressive symptoms in young people.

## Introduction

Mood disorders affect about 20% of the population in lifetime and a quarter of all cases of depression emerge before age 20 (Kessler *et al.* 2003, 2005). The second decade of life is a critical period for brain development; decrease in grey matter volume and increase in connectivity and integrative processing occur during adolescence (Gogtay *et al.* 2004). These developmental events are mirrored by a tide of emergent mental disorders such that, excluding neurodegenerative disorders of later life, about half of all mental disorders first occur by the mid-teens (Kessler *et al.* 2005; Jones, 2013). Distinct trajectories of depressive and anxiety symptoms emerge during early- to mid-teen age years; persistent and severe symptoms during adolescence and adulthood are associated with markers of altered neurodevelopment such as delayed motor milestones (Colman *et al.* 2007). High depressive symptoms during the second decade of life may represent a vulnerability factor for subsequent major depression (Owens *et al.* 2014).

Non-specific and transitory elements of depression are common in youth and early adulthood, crystallising into a clinical syndrome in some but not all of those who experience them (Merikangas *et al.* 2003). Examining depression at a single time point or average levels over time fails to fully account for population heterogeneity because distinct groups of individuals can arrive at the same end point via different pathways. Therefore, longitudinal studies of depressive symptoms over the early-life course including their antecedents and markers for persistence would be helpful for understanding the causes and mechanisms of depression. Compared with a one off measure of psychopathology longitudinal groups derived from repeated measures is less affected by occasional misreporting or temporary fluctuations in a condition. Such approach is also more efficient as they maximise use of partial responses to inform group membership.

In this study, we have used longitudinal population-based data from the Avon Longitudinal Study of Parents and Children (ALSPAC) birth cohort to identify developmental subgroups of young people based on self-reported depressive symptoms measured six times between 10 and 19 years using the Short Mood and Feelings Questionnaire (SMFQ). We have then examined risk factors associated with persistent depressive symptoms particularly focusing on two biological processes, neurodevelopment and inflammation. Longitudinal studies reporting an association with lower childhood IQ indicate a neurodevelopmental component to adult depression (Zammit *et al.* 2004; Koenen *et al.* 2009). We have previously shown that childhood inflammation is associated with increased risks of depressive and psychotic symptoms subsequently in early-adulthood (Khandaker *et al.* 2014*a*). There is some evidence for a link between inflammation, neurodevelopment and cognitive function. Both infection (Khandaker *et al.* 2013) and immunological genes (Sekar *et al.* 2016) associated with the risk of psychosis can interfere with neurodevelopment. Inflammation is associated with reduced cognitive performance in experimental studies of healthy volunteers (Reichenberg *et al.* 2001; Harrison *et al.* 2009) and with cognitive decline in clinical populations at risk of dementia (Iwashyna *et al.* 2010). Although previous longitudinal studies have reported associations between childhood lower IQ, inflammation and depression in adulthood, it is unclear whether these risk factors are associated with persistent symptoms. Furthermore, to our knowledge no study has examined the interplay between inflammation and neurodevelopment with regards to risk of depression.

Using longitudinal population-based data, we have tested the hypothesis that childhood general intelligence, as measured by IQ at 8 years, and inflammation, as measured by elevated serum concentrations of interleukin 6 (IL-6) and C-reactive protein (CRP) at 9 years, are associated with persistent depressive symptoms subsequently between age 10 and 19 years independently of each other. We have explored linearity of association; the effects of past psychological and behavioural problems, initial severity of depressive symptoms, and sex difference on the IQ/inflammation-persistent depressive symptom relationship (see statistical analysis for rationale and approach). In addition, we have examined the relationship between IQ and inflammatory markers. We have tested whether inflammation mediates and/or moderates the relationship between childhood IQ and subsequent persistent depressive symptoms.

## Methods

### Description of cohort and sample

The ALSPAC birth cohort recruited 14 541 pregnant women resident in county Avon, a geographically defined region in southwest of England, with expected dates of delivery 1st April 1991 to 31st December 1992 (http://www.bristol.ac.uk/alspac/) (Boyd *et al.* 2013). The study website contains details of all available data through a fully searchable ‘data dictionary’ (http://www.bris.ac.uk/alspac/researchers/data-access/data-dictionary/). Parents completed regular postal questionnaires about all aspects of their child's health and development from birth. Since age 7, the children attended an annual assessment clinic during which they participated in various face-to-face interviews and physical tests.

Groups of depressive symptoms were created using 9156 participants who had completed the SMFQ at least once between 10 and 19 years. This approach to sample selection – as opposed to including only those who had completed the SMFQ at all six time points – increased representativeness of the sample, sample size and statistical power. Sample size for the analyses of childhood IQ (*N* = 6833) and inflammatory markers (*N* = 4409) vary as these childhood measures were completed by different numbers of people. IQ was assessed on 7354 individuals at 8 years, of which 6833 provided data for depressive symptoms between 10 and 19 years. IL-6 and CRP were measured on 5076 participants at age 9 years, of which 4409 provided data for depressive symptoms between 10 and 19 years. We excluded 491 children who reported an infection around blood collection from the analyses of inflammatory markers because IL6 and CRP levels sharply increase during an infection, which return to normal after illness subsides; we were interested in inflammatory marker levels in healthy individuals.

### Ethical approval

Ethical approval for the study was obtained from the ALSPAC Ethics and Law Committee and the Local Research Ethics Committees.

### Laboratory methods

Data on two archetypal circulating markers of inflammation, CRP (an acute phase protein) and IL-6 (a pleotropic, inflammatory cytokine), were available from the ALSPAC birth cohort. At age 9 years non-fasting blood samples were collected by venepuncture; samples were immediately spun and frozen at −80 °C after collection. IL-6 and CRP levels were assayed in 2008 after a median of 7.5 years in storage with no previous freeze–thaw cycles during this period. IL-6 was measured by enzyme-linked immunosorbent assay (ELISA) (R&D systems, UK), and high sensitivity CRP by automated particle-enhanced immunoturbidimetric assay (Roche UK). All inter-assay coefficients of variation were <5%. In the total sample, IL-6 values ranged from 0.007 to 20.051 pg/mL and CRP values ranged from 0.01 to 45.17 mg/L (32 subjects over 10 mg/L). No other inflammatory markers were measured on blood samples collected at age 9 years. Serum IL-6 and CRP levels were correlated; Pearson's correlation co-efficient based on natural log-transformed values of IL-6 and CRP was 0.45; *p* < 0.001.

### Psychiatric measures

#### Psychological and behavioural problems at age 7

Mothers completed the parental version of the Strengths and Difficulties Questionnaire (SDQ) when the study child was 7. The SDQ is an age appropriate, valid and reliable tool for measuring psychological and behavioural problems in young children (Goodman, 1997). It measures difficulties in four domains (emotional problems, conduct problems, hyperactivity, peer problems), and gives a total difficulties score of 0–40.

#### IQ at age 8

Full scale, verbal and performance IQ were measured by the Wechsler Intelligence Scale for Children, 3rd UK edition (WISC III) (Wechsler *et al.* 1992). A shortened version of the test was applied by trained psychologists, whereby alternate items (always starting with item number 1 in the standard form) were used for all ten subtests with the exception of the coding subtest, which was administered in its standard form. Used successfully in other studies (Stricker *et al.* 1968; Finch & Chihldress, 1975; Khandaker *et al.* 2014*b*), this method provides valid IQ measures that show robust correlations with neurodevelopmental disorders, neurocognitive measures, such as working memory, short-term memory and sociodemographic factors such as social class (Khandaker *et al.* 2014*b*).

#### Depressive symptoms between ages 10 and 19

Depressive symptoms were measured six times using the SMFQ completed by the participants at ages 10, 13, 14, 17, 18 and 19 years (Angold *et al.* 1995). The SMFQ is a validated tool widely used in epidemiological studies (Thapar & McGuffin, 1998; Sharp *et al.* 2006), which includes 13 items covering core symptoms of depression and anxiety experienced in past 2 weeks. Each item is scored zero (not true), one (sometimes true) or two (true) giving a total score of 0–26. We defined individuals with high depressive symptoms at each assessment using total scores of 11 or above because such scores reflect potentially clinically relevant symptom burden. Using this threshold the specificity and sensitivity for a diagnosis of depression is 85 and 60%, respectively (Angold *et al.* 1995), validated against the Diagnostic Interview Schedule for Children (Costello *et al.* 1982).

#### Assessment of covariates

We included age, sex, father's occupation, ethnicity, maternal Edinburgh Post-natal Depression Scale (EPDS) score, SDQ total difficulties score at age 7, total IQ score at age 8 and body mass index (BMI) at age 9 as potential confounders. Age was recorded at the time of assessment of IQ and inflammatory markers, which was used as a continuous variable. Sex was recoded at birth and was coded as a binary variable. Father's occupation was recorded at birth according to the UK Office of National Statistics Classification System (Class I = professionals and higher managerial workers; II = intermediate occupations; IIIa = skilled non-manual occupations; IIIb= skilled manual occupations; IV = partly skilled occupations; V = unskilled occupations), and was coded as a categorical variable (non-manual, i.e. I + II + IIIa, *v.* manual occupations, i.e. IIIb + IV + V). Ethnicity was recorded at birth and was coded as a categorical variable (White *v.* Others). Maternal depression was measured by EPDS at 8-week post-partum and was used as a continuous variable. BMI was recoded at age 9 years at the time of blood collection, which was calculated as weight in kilogram divided by height in metre squared. See above for descriptions of IQ and SDQ.

### Statistical analysis

#### Latent class analysis (LCA) and baseline comparisons

LCA was used to identify classes or population sub-groups based on six longitudinal assessments of depressive symptoms between 10 and 19 years (Collins & Lanza, 2010). Total SMFQ scores from each wave of follow-up were coded as a binary variable using score 11 as cut-off because we were interested in the association between childhood biomarkers and subsequent population sub-groups demarcated by potentially clinically relevant depressive symptom burden. Bayesian information criterion (BIC) was used to assess whether a two, three, or four class model provided a better fit for the data (Schwarz, 1978); the best-fitting model is the one with the lowest BIC. LCA is the stochastic model, so each person does not belong to a class with 100% probability, unlike for example in cluster analysis, rather each person is assigned to the population sub-group they are *most likely* to belong. We examined the relationship between population sub-groups of depressive symptoms and known sociodemographic and other correlates of depression at baseline. Baseline comparisons for IQ were carried out using linear regression to calculate mean difference (95% CI) in full scale, verbal and performance IQ between groups.

#### Association between persistent depressive symptoms and IQ, IL-6 and CRP

We used multinomial logistic regression to calculate odds ratios (OR) for persistent and adolescent-onset depressive symptoms, compared with no symptoms, per s.d. increase in IL-6 levels at baseline. First, we report unadjusted ORs. Second, we report ORs after adjusting for total IQ scores at age 8 years. This is because we wanted to examine whether evidence for association between IL-6 and persistent depressive symptoms remained after taking into account general intelligence. Third, we report ORs after adjusting for childhood psychological and behavioural problems, i.e. SDQ total difficulties score at age 7 years. Childhood psychological and behavioural problems are associated with lower IQ (Horwood *et al.* 2008), higher levels of inflammatory markers (Khandaker *et al.* 2014*a*), and increased risk of depression and other psychiatric disorders subsequently in childhood and adulthood (Rutter *et al.* 2006). Therefore, we wanted to examine whether evidence for association between IL-6 and persistent depressive symptoms remained after taking into account psychological and behavioural problems preceding the measurement of IL-6. Finally, we report ORs after adjusting for all potential confounders, which are age, sex, father's occupation, ethnicity, maternal EPDS score, SDQ total difficulties score, total IQ and BMI (see assessment of covariates for details). We followed the same analytic approach when CRP and IQ were used as predictor. To test whether childhood IQ and IL-6 were independent predictors of subsequent depressive symptoms we controlled the multinomial regression model of IQ for IL-6 and *vice versa*. Linearity of association between IQ and persistent depressive symptoms was tested by including a quadratic term (IQ^2^) in the regression models; the same approach was used for IL-6 using IL-6^2^ as quadratic term.

#### Effect of initial depressive symptom level

Population sub-groups identified by LCA reflect initial differences in depressive symptom severity at age 10 years as well as the subsequent course until age 19 years. To test whether any association with inflammation and IQ pertain to initial difference in symptom severity or subsequent course of depressive symptoms, we controlled multinomial logistic regression models for total SMFQ score at 10 years.

#### Relationship between IQ and inflammatory markers

Linear regression examined the relationship between total IQ and serum IL-6, CRP levels using natural log-transformed values for the inflammatory markers which were normally distributed. We calculated the beta-coefficient and 95% confidence interval for change in serum inflammatory marker levels, in standard deviation, for each standard deviation increase in IQ. Regression models were adjusted for age at blood collection, sex, social class and ethnicity.

#### Moderating and mediating effect of IL-6

We tested for any interaction between IQ and IL-6 by including an interaction term (IQxIL-6) in multinomial logistic regression models using IQ and IL-6 as predictor and persistent depressive symptoms as outcome. We also examined whether IL-6 levels at 9 years mediated the relationship between IQ at 8 years and persistent depressive symptoms at 10–19 years based on sample with data on all three variables. Mediation analysis was carried out using logistic regression (MPlus software); persistent depressive symptoms was used as outcome (binary variable), IQ was used as predictor (continuous variable), and IL-6 was used as mediator (continuous variable). The analyses compared persistent depressive symptoms with no symptoms; participants with adolescent-onset symptoms were excluded. We calculated regression co-efficient and standard error for direct and indirect effect of IQ on risk of persistent depressive symptoms. Presence of a significant indirect effect would indicate mediation of association between IQ and persistent depressive symptoms by IL-6.

#### Sex difference

We examined whether sex differences applied to the association between the adolescent depressive sub-groups and childhood biomarkers. First, we calculated the ORs for persistent depressive symptoms for each childhood biomarker separately for two sexes using multinomial regression as above. Second, we included a sex × biomarker (separately for IQ and IL-6) interaction term in the multinomial regression models based on total sample.

## Results

### Population sub-groups of depressive symptoms between 10 and 19 years

Total SMFQ score and the percentage of subjects with a high burden of depressive symptoms (total SMFQ score 11 or above) increased with age between 10 and 19 years (Table 1).

**Table 1.** Total SMFQ Score between ages 10 and 19 years in the ALSPAC cohort

Age at assessment (years)	Total no.	SMFQ score 11 or above, no. (%)	SMFQ median (IQR)			
			All subjects	Male	Female	*p* value^a^
10	7367	441 (6.0)	3 (1–6)	3 (2–6)	3 (1–6)	<0.001
13	6682	472 (5.2)	3 (1–5)	3 (1–5)	3 (1–6)	<0.001
14	6020	703 (11.7)	4 (2–7)	3 (1–6)	4 (2–8)	<0.001
17	4994	902 (18.1)	4 (2–8)	3 (1–6)	5 (2–10)	<0.001
18	4493	973 (21.7)	5 (3–10)	4 (2–8)	6 (3–11)	<0.001
19	3332	730 (21.9)	5 (2–10)	4 (2–7)	6 (3–11)	<0.001

a Independent sample Kruskal–Wallis test used to compare distribution of SMFQ scores between male and female groups.

**Fig. 1.**
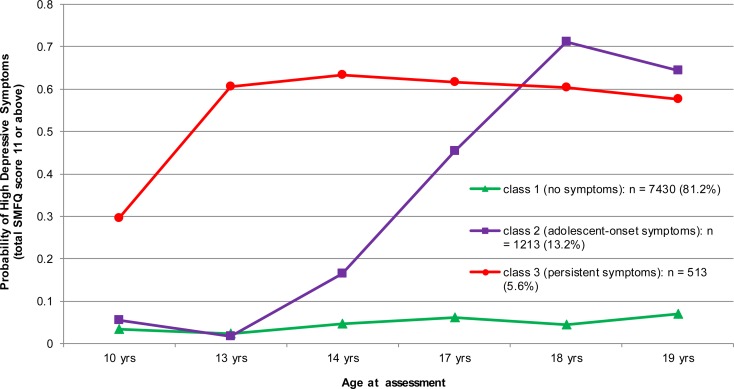
Three Class Model of Depressive Symptoms between 10 and 19 years in the ALSPAC cohort. Note: Class membership is driven by the probability of high depressive symptoms, defined as total SMFQ score 11 or above, at six follow-up points. Class 1 = consistently low symptoms at all six follow-ups (‘No symptoms’). Class 2 = symptoms low in the first two follow-ups but increase subsequently (‘Adolescent-onset symptoms’). Class 3 = consistently high symptoms at all six follow-ups (‘Persistent symptoms’).

After latent class analysis of longitudinal data from 9156 participants a three class model was chosen based on the BIC (Fig. 1). These groups were driven by the probability of high depressive symptoms, defined as SMFQ score 11 or above, at the six follow-up points. The BIC for a two latent class model was 22 582, for a three class model was 22 483, and for a four class model was 22 500.

In the three-class model, class one had consistently low depressive symptoms at all six follow-ups between 10 and 19 years (hereafter, ‘no symptoms’). In class two depressive symptoms were low in the first two follow-ups at 10 and 13 years but increased subsequently (hereafter, ‘adolescent-onset symptoms’). Class three had consistently high depressive symptoms at all six follow-ups (hereafter ‘persistent symptoms’). Therefore, the groups represent individuals defined by consistently low levels of depressive symptoms, occasionally high levels, and consistently high levels of depressive symptoms.

### Baseline sociodemographic characteristics and population sub-groups of depressive symptoms

Sociodemographic characteristics, distributions of maternal EPDS score at 8 weeks postpartum, SDQ score at age 7, IQ and inflammatory markers for the total sample have been presented in Table 2. The population sub-groups of depressive symptoms differed significantly from each other in known sociodemographic and other correlates of depression. Compared with the group with no symptoms, the groups with depressive symptoms were more likely to comprise women, had lower socio-economic background, higher SDQ score at 7 years, and higher maternal post-natal depression score (Table 2).

**Table 2.** Sociodemographic and other correlates of depressive symptoms

Characteristic	Total Sample	Population sub-groups of depressive symptoms between 10 and 19 years			*p* Value^a^
		No symptoms	Adolescent-onset symptoms	Persistent symptoms	
Total no. (%)	9156 (100)	7430 (81.2)	1213 (13.2)	513 (5.6)	–
Male sex, no. (%)	4411 (48.2)	3871 (52.1)	363 (29.9)	177 (34.5)	<0.0001
British White, no. (%)	8059 (97.9)	6534 (97.9)	1077 (98.1)	448 (98.0)	0.92
Father's non-manual occupation, no. (%)	4527 (49.4)	3713 (60.6)	596 (59.4)	218 (53.0)	0.009
Mother's EPDS score, mean (s.d.)	5.89 (4.65)	5.7 (4.60)	6.5 (4.82)	7.0 (4.77)	<0.0001
SDQ total score at age 7 years, mean (s.d.)	7.40 (4.75)	7.2 (4.62)	8.1 (4.99)	9.4 (5.55)	<0.0001
Age at IL-6/CRP assay, mean (s.d.) (days)	3616 (115.6)	3617 (115.1)	3612 (117.3)	3622 (118.9)	0.27
BMI at IL-6 and CRP assay, mean (s.d.)	17.7 (2.90)	17.6 (2.84)	17.9 (3.10)	18.0 (3.23)	0.003
IQ at 8 years					
Total no. (%)	6833 (100)	5578 (81.6)	862 (12.6)	393 (5.75)	–
Full scale IQ, mean (s.d.)	104.63 (16.38)	105.02 (16.40)	104.19 (15.51)	100.09 (17.25)	<0.0001
Verbal IQ, mean (s.d.)	107.57 (16.71)	107.97 (16.78)	107.02 (16.65)	102.90 (17.28)	<0.0001
Performance IQ, mean (s.d.)	100.02 (16.99)	100.30 (16.98)	99.96 (16.36)	96.53 (18.04)	<0.0001
Serum IL-6 at 9 years (pg/mL)					
Total no. (%)	4409 (100)	3584 (81.28)	559 (12.67)	266 (6.03)	–
Mean (s.d.)		1.18 (1.42)	1.25 (1.49)	1.48 (2.02)	0.005
Median (IQR)		0.76 (0.47–1.31)	0.80 (0.48–1.46)	0.89 (0.55–1.56)	0.017
Serum CRP at 9 years (mg/L)					
Total no. (%)	4417 (100)	3592 (81.32)	559 (12.65)	266 (6.02)	–
Mean (s.d.)		0.58 (1.68)	0.77 (3.00)	0.75 (1.84)	0.053
Median (IQR)		0.20 (0.11–0.47)	0.21 (0.11–0.48)	0.26 (0.12–0.59)	0.045

s.d., standard deviation; SDQ, Strengths and Difficulties Questionnaire ; BMI, body mass index; IQR, interquartile range; pg/mL, picogram per millilitre; mg/L, milligram per litre.

a One way analysis of variance for continuous data (age, mother's depression score, SDQ score, body mass index and mean values of IQ, IL-6 and CRP); Chi-squared test for proportions (male sex, British White ethnicity and father's occupation); independent samples Kruskal–Wallis test for IL-6 and CRP distributions (median).

### Association between IQ at age 8 years and persistent depressive symptoms from 10 to 19 years

Childhood IQ was associated with subsequent persistent (but not adolescent-onset) depressive symptoms. The risk of being in the persistent depressive symptoms group between 10 and 19 years relative to the no symptoms group was 27% lower for each 1-s.d. increase in IQ at 8 years. After adjusting for potential confounders the association attenuated slightly, but evidence for an association remained (Table 3; online Supplementary Fig. S1). See online Supplementary Table S1 for fully adjusted model showing ORs for each covariate. The quadratic term (IQ^2^) in the multinomial regression model was not significant (*p* = 1.00) suggesting a linear dose-response relationship between childhood IQ and subsequent persistent depressive symptoms. The association between IQ and persistent depressive symptoms remained significant after controlling for IL-6, and *vice versa* (see Tables 3 and 4), suggesting that IQ and IL-6 are independent predictors of subsequent persistent depressive symptoms.

**Table 3.** ORs for persistent and adolescent-onset depressive symptoms between 10 and 19 years for each s.d. increase in total IQ score at age 8 years

Group	No. (%)	OR (95% CI)			
		Unadjusted analysis	Adjusted for IL-6 levels at 9 years only	Adjusted for SDQ total difficulties score at 7 years only	Adjusted for age at IQ assessment, sex, father's occupation, ethnicity, maternal EPDS score, SDQ total difficulties score and IL-6 level^a^
No symptoms	5578 (81.6)	1.00 [reference]	1.00 [reference]	1.00 [reference]	1.00 [reference]
Adolescent-onset symptoms	862 (12.6)	0.95 (0.88–1.02)	0.94 (0.86–1.04)	0.98 (0.90–1.06)	0.97 (0.87–1.09)
Persistent symptoms	393 (5.8)	0.73 (0.66–0.81)	0.70 (0.61–0.80)	0.83 (0.73–0.93)	0.80 (0.68–0.95)

a Analysis was controlled for age at assessment of IQ, sex at birth, father's occupational status at birth, ethnicity at birth, maternal Edinburgh Post-natal Depression Scale (EPDS) total score at 8-week post-partum, Strengths and Difficulties Questionnaire (SDQ) total difficulties score at age 7 years, serum IL-6 concentration at age 9 years. Please see section ‘assessment of covariates’ for more information.

**Table 4.** ORs for Persistent and adolescent-onset depressive symptoms between 10 and 19 years for each s.d. increase in IL-6, CRP at age 9 years

Group	No. (%)	OR (95% CI)			
		Unadjusted analysis	Adjusted for total IQ score at 8 years only	Adjusted for SDQ total difficulties score at 7 years only	Adjusted for age at IL-6 assessment, sex, father's occupation, ethnicity, maternal EPDS score, SDQ total difficulties score, IQ, BMI at 9 years^a^
Predictor: per s.d. increase in serum IL-6 at 9 years					
No symptoms	3584 (81.3)	1.00 [reference]	1.00 [reference]	1.00 [reference]	1.00 [reference]
Adolescent-onset symptoms	559 (12.7)	1.05 (0.96–1.15)	1.03 (0.93–1.14)	1.08 (0.98–1.20)	1.08 (0.95–1.22)
Persistent symptoms	266 (6.0)	1.18 (1.06–1.31)	1.16 (1.04–1.30)	1.17 (1.04–1.33)	1.20 (1.03–1.39)
Predictor: per s.d. increase in serum CRP at 9 years					
No symptoms	3592 (81.3)	1.00 [reference]	1.00 [reference]	1.00 [reference]	1.00 [reference]
Adolescent-onset symptoms	559 (12.7)	1.11 (1.01–1.23)	1.09 (0.97–1.23)	1.11 (1.01–1.24)	1.14 (0.97–1.34)
Persistent symptoms	266 (6.0)	1.10 (0.96–1.27)	1.08 (0.91–1.27)	1.09 (0.93–1.26)	1.16 (0.95–1.43)

a Analysis was controlled for age at assessment of IL-6, sex at birth, father's occupational status at birth, ethnicity at birth, maternal Edinburgh Post-natal Depression Scale (EPDS) total score at 8-week post-partum, Strengths and Difficulties Questionnaire (SDQ) total difficulties score at age 7 years, total IQ score at 8 years, and BMI at age 9 years. Please see section ‘assessment of covariates’ for more information.

### Association between IL-6 and CRP levels at age 9 years and persistent depressive symptoms from 10 to 19 years

Higher IL-6 levels at 9 years were associated with subsequent persistent depressive symptoms between 10 and 19 years. The adjusted OR for being in the persistent depressive symptoms group relative to the no symptoms group for each 1-s.d. increase in IL-6 levels was 1.20 (95% CI, 1.03–1.39) (Table 4; online Supplementary Fig. S1). The quadratic term (IL-6^2^) in the multinomial regression model was not significant (*p* = 0.80) suggesting a linear dose–response relationship between childhood IL-6 levels and subsequent persistent depressive symptoms. There was no evidence for an association between CRP and persistent depressive symptoms. See online Supplementary Tables S2 and S3 for fully adjusted models for IL-6 and CRP showing ORs for each covariate.

### Effect of psychological and behavioural problems at age 7 years

Evidence of associations between childhood IQ at 8 years, IL-6 at 9 years and subsequent persistent depressive symptoms at 10–19 years all persisted after adjusting for SDQ score at age 7 (Tables 3 and 4), suggesting that the associations between persistent depressive symptoms, childhood IQ and IL-6 levels were independent of the effect of past psychological and behavioural problems on these biomarkers.

### Effect of initial depressive symptom level at age 10 years

The OR for persistent depressive symptoms between 10 and 19 years for childhood IL-6 at 9 years remained significant after controlling for total SMFQ score at 10 years; adjusted OR = 1.16 (95% CI, 1.03–1.31); *p* = 0.018. This suggests that childhood IL-6 levels at 9 years predict the course of depressive symptoms between 10 and 19 years independently of initial symptom severity at age 10 years. However, the OR for persistent depressive symptoms from 10 to 19 years for childhood IQ at 8 years became non-significant after controlling for total SMFQ score at 10 years; adjusted OR = 0.95 (95% CI, 0.85–1.08); *p* = 0.484.

### Association between total IQ and serum IL-6, CRP level

Higher total IQ scores at age 8 years were associated with lower serum CRP levels at age 9 years; *β* = −0.071 (95% CI, −1.00, −0.041); *p* < 0.001. Evidence for this association remained after adjusting for age, sex, father's occupation and ethnicity; adjusted *β* = −0.048 (95% CI, −0.080, −0.016); *p* = 0.003. Similarly, higher total IQ scores at age 8 years was associated with lower serum IL-6 levels at age 9 years; *β* = −0.046 (95% CI, −0.077, −0.015); *p* = 0.004. However, this association was attenuated after adjusting for age, sex, father's occupation and ethnicity; adjusted *β* = −0.024 (95% CI, −0.050, 0.010); *p* = 0.163.

### Moderating and mediating effects of IL-6 on the IQ-persistent depressive symptoms relationship

There was no evidence for any interaction between IQ and IL-6 in relation to risk of persistent depressive symptoms (*p* value for interaction term = 0.149). With regards to results of mediation analysis, based on 3350 participants the direct effect of IQ on persistent depressive symptoms was significant indicating an association between IQ and persistent depressive symptoms; regression coefficient for direct effect = −0.188, s.e. = 0.037; *p* < 0.001. However, there was no evidence that IL-6 at 9 years mediated the relationship between IQ at 8 years and persistent depressive symptoms between 10 and 19 years; regression co-efficient for indirect effect = −0.004, s.e. = 0.002; *p* = 0.073. The regression coefficient for total effect (a sum of indirect and direct effect) was −0.192, s.e. = 0.037; *p* < 0.001.

### Sex difference in the association between IQ, IL-6 and persistent depressive symptoms

In analyses stratified by sex, childhood IQ was associated with subsequent persistent depressive symptoms in both sexes (see online Supplementary Table S4). For IL-6, although the ORs were similar between sexes that for men was no longer statistically significant. However, no tests for interaction between IQ and sex and IL-6 and sex in the respective regression models approached statistical significance (both *p* > 0.10), suggesting no significant sex difference in the overserved associations between IQ, IL-6 and persistent depressive symptoms.

## Discussion

Using population-based longitudinal birth cohort data, we defined a population sub-group of young people characterised by persistent depressive symptoms during the second decade of life. Lower IQ at 8 years, a measure of neurodevelopment, and higher IL-6 at 9 years, a measure of inflammation, were independently associated with increased risk of subsequent persistent (but not adolescent-onset) depressive symptoms between 10 and 19 years in a linear, dose–response fashion. Evidence for an association persisted after controlling for a number of potential confounders including sex, body mass, social class, ethnicity, maternal post-natal depression and psychological and behavioural problems measured before the biomarkers at 7 years. To our knowledge this is one of the first longitudinal studies of childhood IQ, inflammatory markers and subsequent persistent depressive symptoms. The results are consistent with potentially important roles for inflammation and neurodevelopment in the aetiology of persistent depressive symptoms, and indicate important differences between adolescent-onset and persistent depressive symptoms during the second decade of life.

Findings from the current study are consistent with previous longitudinal studies reporting an association between adult depression and lower IQ in childhood (van Os *et al.* 1997; Zammit *et al.* 2004; Koenen *et al.* 2009). The results are also consistent with previous longitudinal studies showing an association between adult depression, higher IL-6 levels in childhood (Khandaker *et al.* 2014*a*) and in adulthood (Zalli *et al.* 2016). Poorer childhood IQ has been associated with increased risk of adult schizophrenia (Jones *et al.* 1994; David *et al.* 1997; Davidson *et al.* 1999; Cannon *et al.* 2000; Cannon *et al.* 2002; Woodberry *et al.* 2008; Khandaker *et al.* 2011), post-traumatic stress disorder (Koenen *et al.* 2007; Kremen *et al.* 2007), anxiety (Koenen *et al.* 2009) and substance misuse disorders (Mortensen *et al.* 2005). It has been suggested that general cognitive ability as measured by IQ may be a marker of the integrity of the nervous system (Whalley & Deary, 2001) and of cognitive reserve (Barnett *et al.* 2006), which might underlie the association between childhood IQ and subsequent risk of mental illness. Association with lower IQ indicates a neurodevelopmental component to depressive symptoms persistent during late childhood and teenage years.

Accumulating evidence implicates the immune system, particularly low-grade systemic inflammation defined by elevated concentrations of circulating inflammatory markers, in the pathogenesis of depression, psychosis and other major mental disorders (Raison *et al.* 2006; Dantzer *et al.* 2008; Khandaker *et al.* 2015). CRP is an archetypal acute phase protein and IL-6 is a pleotropic proinflammatory cytokine, both of which have been studied extensively in depression and shown to be elevated in acutely unwell patients compared with controls (Howren *et al.* 2009; Haapakoski *et al.* 2015; Goldsmith *et al.* 2016). Longitudinal studies including our own have shown that elevated IL-6 or CRP levels are associated with increased risks of developing depression and psychosis in future (Gimeno *et al.* 2009; Wium-Andersen *et al.* 2014; Khandaker *et al.* 2014*a*; Metcalf *et al.* 2017). The current study adds to the existing evidence by showing that childhood IL-6 levels are associated with subsequent persistent depressive symptoms, which might indicate that low-grade systemic inflammation contribute to development and persistence of depressive symptoms.

The current study is distinct from previous studies from the ALSPAC birth cohort that have examined the relationship between inflammation and psychiatric symptoms. Childhood IL-6 levels at 9 years have been reported to be associated with atopic disorders (Khandaker *et al.* 2014*c*), depressive and psychotic symptoms assessed at 18 years (Khandaker *et al.* 2014*a*), and hypomanic symptoms assessed at age 22 years (Hayes *et al.* 2016). Another study has reported association between serum CRP levels and generalised anxiety disorder both assessed at age 15 years (Khandaker *et al.* 2016). None of the previous studies concerned persistent depressive symptoms between ages 10 and 19.

The use of a general population birth cohort, relatively large sample, and prospective assessments for depressive symptoms, IQ and inflammatory markers are some of the strengths of the study. Limitations include missing data. The number of respondents with missing data for SMFQ increased in successive follow-ups between 10 and 19 years. Only about 1700 participants completed the questionnaire at all six follow-up points, a group unlikely to be representative of the general population. Therefore, in order to avoid any bias from missing data we included individuals who completed at least one out of six SMFQ questionnaires between 10 and 19 years for the calculation of latent classes. In constructing the latent classes of depressive symptoms we deliberately used dichotomised SMFQ scores at six follow-up points as opposed to SMFQ total scores or item level data. This is because we were interested in the evolution of clinically relevant depressive symptoms during critical period of brain development; the threshold used to dichotomise SMFQ data (total score 11 or above) has been reported to represent burden of depressive symptoms that may be clinically relevant (Angold *et al.* 1995).

We did not have any data on depression at the time of IQ or IL-6 measurements at ages 8 and 9 years, respectively, so the possibility of reverse causality cannot be ruled out. It is plausible that persistent depressive symptoms between ages 10 and 19 represent continuation of depressive symptoms that started earlier in childhood before the assessment of the biomarkers. However, the results remained significant after controlling for SDQ score at 7 years providing some reassurance that reverse causality is unlikely to be the sole explanation for the observed results.

We acknowledge any diurnal variation in serum IL-6 and CRP concentrations may not be adequately captured by non-fasting blood samples (de Jager *et al.* 2009). However, the use of non-fasting samples would increase measurement error likely to be random in relation to the outcome. No other blood samples from childhood or adolescence were available to examine long-term within-individual consistency of inflammatory marker levels. However, one study of healthy volunteers found no significant changes in serum IL-6 levels over a 3-year period (Knudsen *et al.* 2008). Similarly, a large meta-analysis of population-based studies of coronary heart disease has found that values for CRP are sufficiently stable over time (Danesh *et al.* 2004). There was some evidence that higher IQ at baseline was associated with subsequent lower inflammatory marker levels. However, an opposite effect, i.e. inflammation influencing cognitive function in young people, is also plausible. In future prospective studies with data on inflammatory markers at baseline and cognitive function in follow-up are needed to examine this hypothesis. Childhood and adolescent infection/inflammation affect adolescent neurodevelopment, which is further complicated by malnutrition (Galler *et al.* 2017; John *et al.* 2017). While this is particularly relevant for low and middle-income countries, and that serious malnutrition is unlikely to be a major issue in a contemporary UK cohort, in future studies should examine the relationship between inflammation, nutrition and development.

In summary, we report one of the first longitudinal studies of childhood IQ, inflammatory markers and subsequent persistent depressive symptoms during the second decade of life. The findings indicate important roles for two distinct biological processes, inflammation and neurodevelopment, in the aetiology of persistent depressive symptoms.

## Supplementary material

For supplementary material accompanying this paper visit https://doi.org/10.1017/S0033291717003038. click here to view supplementary material
