# Supplementary material for: Childhood inflammatory markers and intelligence as predictors of subsequent persistent depressive symptoms: a longitudinal cohort study
Source: Psychol Med. 2017 Nov 15;48(9):1514–22. doi: 10.1017/S0033291717003038 (PMC6088526; doi:10.1017/S0033291717003038)
Supplement: Supplementary file 1 [file S0033291717003038sup001.docx]

**Online Supplementary Material**

**Khandaker *et al*. Childhood Inflammatory Markers and Intelligence as Predictors of Subsequent Persistent Depressive Symptoms: A Longitudinal Cohort Study**

**Online Supplementary Table 1: Adjusted ORs for Persistent and Adolescent-onset Depressive Symptoms between Ages 10 and 19 Years for each SD Increase in IQ at Age 8 Years – Table showing fully adjusted model with ORs for each variable**

**(Predictor IQ in yellow highlight)**

| **Parameter Estimates** | | | | | | | | | |
| --- | --- | --- | --- | --- | --- | --- | --- | --- | --- |
| MFQ latent categories from 10 to 18 yrs^a^ | | B | Std. Error | Wald | df | P-value | OR | 95% Confidence Interval for OR | |
|  |  |  |  |  |  |  |  | Lower Bound | Upper Bound |
| Adolescent-onset symptoms | Intercept | -.264 | 2.264 | .014 | 1 | .907 |  |  |  |
|  | IQ at 8y | -.026 | .059 | .191 | 1 | .662 | .975 | .869 | 1.093 |
|  | SDQ at 7y | .041 | .012 | 11.854 | 1 | .001 | 1.041 | 1.018 | 1.066 |
|  | IL6_at 9y | .027 | .035 | .617 | 1 | .432 | 1.028 | .960 | 1.100 |
|  | EPDS score | .029 | .012 | 6.204 | 1 | .013 | 1.030 | 1.006 | 1.054 |
|  | Age at IQ | -.001 | .001 | 1.027 | 1 | .311 | .999 | .998 | 1.001 |
|  | Sex [male=1] | -.905 | .112 | 65.362 | 1 | .000 | .405 | .325 | .504 |
|  | Sex [female=2] | 0^b^ | . | . | 0 | . | . | . | . |
|  | Ethnicity [white=1] | .461 | .537 | .738 | 1 | .390 | 1.586 | .554 | 4.540 |
|  | Ethnicity [non-white=2] | 0^b^ | . | . | 0 | . | . | . | . |
|  | Father’s occupation [non-manual=1] | .151 | .116 | 1.689 | 1 | .194 | 1.163 | .926 | 1.461 |
|  | Father’s occupation [manual=2] | 0^b^ | . | . | 0 | . | . | . | . |
| Persistent symptoms | Intercept | -5.096 | 2.735 | 3.473 | 1 | .062 |  |  |  |
|  | IQ at 8y | -.215 | .085 | 6.319 | 1 | .012 | .807 | .682 | .954 |
|  | SDQ at 7y | .088 | .015 | 32.356 | 1 | .000 | 1.092 | 1.059 | 1.125 |
|  | IL6_at 9y | .089 | .043 | 4.288 | 1 | .038 | 1.093 | 1.005 | 1.188 |
|  | EPDS score | .037 | .016 | 5.111 | 1 | .024 | 1.038 | 1.005 | 1.071 |
|  | Age at IQ | .001 | .001 | .619 | 1 | .431 | 1.001 | .999 | 1.002 |
|  | Sex [male=1] | -.925 | .165 | 31.457 | 1 | .000 | .396 | .287 | .548 |
|  | Sex [female=2] | 0^b^ | . | . | 0 | . | . | . | . |
|  | Ethnicity [white=1] | -.291 | .548 | .282 | 1 | .596 | .748 | .255 | 2.189 |
|  | Ethnicity [non-white=2] | 0^b^ | . | . | 0 | . | . | . | . |
|  | Father’s occupation [non-manual=1] | -.071 | .164 | .190 | 1 | .663 | .931 | .676 | 1.283 |
|  | Father’s occupation [manual=2] | 0^b^ | . | . | 0 | . | . | . | . |

**Online Supplementary Table 2: Adjusted ORs for Persistent and Adolescent-onset Depressive Symptoms between Ages 10 and 19 Years for each SD Increase in IL-6 at Age 9 Years – Table showing fully adjusted model with ORs for each variable**

**(Predictor IL-6 in yellow highlight)**

| **Parameter Estimates** | | | | | | | | | |
| --- | --- | --- | --- | --- | --- | --- | --- | --- | --- |
| MFQ latent categories from 10 to 18 yrs^a^ | | B | Std. Error | Wald | df | P-value | OR | 95% Confidence Interval for OR | |
|  |  |  |  |  |  |  |  | Lower Bound | Upper Bound |
| Adolescent-onset symptoms | Intercept | .328 | 2.200 | .022 | 1 | .881 |  |  |  |
|  | IL6_at 9y | .078 | .062 | 1.566 | 1 | .211 | 1.081 | .957 | 1.222 |
|  | IQ at 8y | -.017 | .062 | .075 | 1 | .785 | .983 | .870 | 1.111 |
|  | SDQ at 7y | .039 | .012 | 9.925 | 1 | .002 | 1.040 | 1.015 | 1.065 |
|  | EPDS at 8wk | .038 | .012 | 9.285 | 1 | .002 | 1.039 | 1.014 | 1.064 |
|  | BMI at 9y | -.004 | .021 | .044 | 1 | .834 | .996 | .956 | 1.037 |
|  | Age at IL-6 assay | -.001 | .001 | 1.850 | 1 | .174 | .999 | .998 | 1.000 |
|  | Sex [male=1] | -.872 | .117 | 55.209 | 1 | .000 | .418 | .332 | .526 |
|  | Sex [female=2] | 0^b^ | . | . | 0 | . | . | . | . |
|  | Ethnicity [white=1] | .574 | .615 | .870 | 1 | .351 | 1.775 | .532 | 5.924 |
|  | Ethnicity [non white=2] | 0^b^ | . | . | 0 | . | . | . | . |
|  | Father’s occupation [non manual=1] | .173 | .123 | 2.002 | 1 | .157 | 1.189 | .935 | 1.512 |
|  | Father’s occupation [manual=2] | 0^b^ | . | . | 0 | . | . | . | . |
| Persistent symptoms | Intercept | .219 | 3.120 | .005 | 1 | .944 |  |  |  |
|  | IL6 at 9y | .183 | .077 | 5.581 | 1 | .018 | 1.200 | 1.032 | 1.397 |
|  | IQ at 8y | -.274 | .092 | 8.935 | 1 | .003 | .760 | .635 | .910 |
|  | SDQ at 7y | .089 | .016 | 29.878 | 1 | .000 | 1.093 | 1.059 | 1.128 |
|  | EPDS at 8wk | .039 | .017 | 5.086 | 1 | .024 | 1.040 | 1.005 | 1.076 |
|  | BMI at 9y | .003 | .029 | .010 | 1 | .921 | 1.003 | .947 | 1.062 |
|  | Age at IL-6 assay | -.001 | .001 | 1.014 | 1 | .314 | .999 | .997 | 1.001 |
|  | Sex [male=1] | -.832 | .173 | 23.027 | 1 | .000 | .435 | .310 | .611 |
|  | Sex [female=2] | 0^b^ | . | . | 0 | . | . | . | . |
|  | Ethnicity [white=1] | -.441 | .558 | .624 | 1 | .430 | .644 | .216 | 1.921 |
|  | Ethnicity [non white=2] | 0^b^ | . | . | 0 | . | . | . | . |
|  | Father’s occupation [non manual=1] | -.023 | .174 | .017 | 1 | .895 | .977 | .695 | 1.375 |
|  | Father’s occupation [manual=2] | 0^b^ | . | . | 0 | . | . | . | . |

| a. The reference category is: No sympytoms. |
| --- |
| b. This parameter is set to zero because it is redundant. |

**Online Supplementary Table 3: Adjusted ORs for Persistent and Adolescent-onset Depressive Symptoms between Ages 10 and 19 Years for each SD Increase in CRP at Age 9 Years – Table showing fully adjusted model with ORs for each variable**

**(Predictor CRP in yellow highlight)**

| **Parameter Estimates** | | | | | | | | | |
| --- | --- | --- | --- | --- | --- | --- | --- | --- | --- |
| MFQ latent categories from 10 to 18 yrs^a^ | | B | Std. Error | Wald | df | P-value | OR | 95% Confidence Interval for OR | |
|  |  |  |  |  |  |  |  | Lower Bound | Upper Bound |
| Adolescent-onset symptoms | Intercept | .113 | 2.196 | .003 | 1 | .959 |  |  |  |
|  | CRP at 9y | .137 | .083 | 2.694 | 1 | .101 | 1.146 | .974 | 1.349 |
|  | IQ at 8y | -.010 | .062 | .027 | 1 | .870 | .990 | .876 | 1.119 |
|  | SDQ at 7y | .040 | .012 | 10.204 | 1 | .001 | 1.040 | 1.015 | 1.066 |
|  | EPDS at 8wk | .038 | .012 | 9.200 | 1 | .002 | 1.038 | 1.013 | 1.064 |
|  | BMI at 9y | -.007 | .021 | .110 | 1 | .740 | .993 | .953 | 1.035 |
|  | Age at IL-6 assay | -.001 | .001 | 1.494 | 1 | .222 | .999 | .998 | 1.000 |
|  | Sex [male=1] | -.872 | .117 | 55.199 | 1 | .000 | .418 | .332 | .526 |
|  | Sex [female=2] | 0^b^ | . | . | 0 | . | . | . | . |
|  | Ethnicity [white=1] | .546 | .615 | .787 | 1 | .375 | 1.726 | .517 | 5.765 |
|  | Ethnicity [non white=2] | 0^b^ | . | . | 0 | . | . | . | . |
|  | Father’s occupation [non manual=1] | .164 | .123 | 1.794 | 1 | .180 | 1.179 | .927 | 1.499 |
|  | Father’s occupation [manual=2] | 0^b^ | . | . | 0 | . | . | . | . |
| Persistent symptoms | Intercept | -.022 | 3.106 | .000 | 1 | .994 |  |  |  |
|  | CRP at 9y | .155 | .105 | 2.189 | 1 | .139 | 1.168 | .951 | 1.435 |
|  | IQ at 8y | -.272 | .092 | 8.772 | 1 | .003 | .762 | .636 | .912 |
|  | SDQ at 7y | .089 | .016 | 30.022 | 1 | .000 | 1.093 | 1.059 | 1.128 |
|  | EPDS at 8wk | .040 | .017 | 5.285 | 1 | .022 | 1.041 | 1.006 | 1.077 |
|  | BMI at 9y | .005 | .029 | .027 | 1 | .870 | 1.005 | .949 | 1.064 |
|  | Age at IL-6 assay | -.001 | .001 | .834 | 1 | .361 | .999 | .998 | 1.001 |
|  | Sex [male=1] | -.831 | .173 | 22.950 | 1 | .000 | .436 | .310 | .612 |
|  | Sex [female=2] | 0^b^ | . | . | 0 | . | . | . | . |
|  | Ethnicity [white=1] | -.518 | .555 | .870 | 1 | .351 | .596 | .201 | 1.769 |
|  | Ethnicity [non white=2] | 0^b^ | . | . | 0 | . | . | . | . |
|  | Father’s occupation [non manual=1] | -.034 | .174 | .039 | 1 | .843 | .966 | .687 | 1.359 |
|  | Father’s occupation [manual=2] | 0^b^ | . | . | 0 | . | . | . | . |

| a. The reference category is: No symptoms. |
| --- |
| b. This parameter is set to zero because it is redundant. |

**Online Supplementary Table 4: Sex-Stratified ORs for Persistent Depressive Symptoms between Ages 10 and 19 Years per SD Increase in Childhood Biomarker Levels**

| **Predictors** | **Male** | | **Female** | |
| --- | --- | --- | --- | --- |
|  | **No. (%)** | **OR (95% CI)** | **Total No. (%)** | **OR (95% CI)** |
| **IL-6 at 9 Years** |  |  |  |  |
| No symptoms | 1956 (87.8) | 1.00 [reference] | 1628 (74.7) | 1.00 [reference] |
| Adolescent-onset symptoms | 185 (8.3) | 1.00 (0.85-1.18) | 374 (17.1) | 1.04 (0.92-1.17) |
| Persistent symptoms | 88 (3.9) | 1.11 (0.92-1.34) | 178 (8.2) | 1.18 (1.04-1.35) |
| **CRP at 9 Years** |  |  |  |  |
| No symptoms | 1962 (87.8) | 1.00 [reference] | 1630 (74.7) | 1.00 [reference] |
| Adolescent-onset symptoms | 185 (8.3) | 0.91 (0.65-1.25) | 374 (17.1) | 1.14 (1.01-1.29) |
| Persistent symptoms | 88 (3.9) | 1.03 (0.76-1.39) | 178 (8.2) | 1.11 (0.94-1.32) |
| **Full scale IQ at 8 Years** |  |  |  |  |
| No symptoms | 2947 (88.1) | 1.00 [reference] | 2631 (75.5) | 1.00 [reference] |
| Adolescent-onset symptoms | 265 (7.9) | 1.04 (0.92-1.17) | 597 (17.1) | 0.91 (0.82-0.99) |
| Persistent symptoms | 134 (4.0) | 0.70 (0.58-0.82) | 259 (7.4) | 0.75 (0.66-0.86) |
